# Sugars, Alcohol, and Caffeine Intake From Drinks Among Outpatients With Mental Health Disorders in Greece: A Pilot Study

**DOI:** 10.7759/cureus.21563

**Published:** 2022-01-24

**Authors:** Xenia A Apostolakopoulou, Lamprini Kontopoulou, Georgios E Karpetas, Georgios Marakis, Eleni Vasara, Ioannis G Katsaras, Zoi Maraki, Ioanna V Papathanasiou, Konstantinos S Bonotis

**Affiliations:** 1 Department of Nutrition, University of Thessaly, Larissa, GRC; 2 Department of Nursing, University of Thessaly, Larissa, GRC; 3 Department of Medicine, University of Thessaly, Larissa, GRC; 4 Nutrition and Food Standards Unit, Hellenic Food Authority, Athens, GRC; 5 Department of Zoology, School of Biology, Aristotle University of Thessaloniki, Thessaloniki, GRC; 6 New Vitality Clinic, Reading Clinic, Reading, GBR

**Keywords:** affective disorders, schizophrenia, mental disorder, caffeine, alcohol, sugar, obesity

## Abstract

Background and aim

Excessive intake of sugars and energy from drinks has been postulated to increase the risk of obesity, which may in turn be associated with mental health disorders. In addition, excessive intakes of alcohol and caffeine may co-occur with psychiatric disorders. The purpose of the present pilot study was to estimate energy, sugar, caffeine, and alcohol intakes through the consumption of drinks in patients with schizophrenia and affective disorders and assess potential differences in drink consumption between the two disorders.

Methodology

The current study included 89 outpatients with schizophrenia (n = 36) and affective disorders (n = 53) attending the psychiatric clinic of the University General Hospital of Larissa (UGHL) in Greece. In addition to anthropometric measurements, the patients were asked to complete a specific, previously validated questionnaire on the frequency of drink consumption in order to estimate sugar, caffeine, and alcohol intakes.

Results

The participants had a mean body mass index (BMI) of 28.9 ± 5.6 kg/m2 without significant differences between the two types of mental disorders. Similarly, the mean waist circumference (102.6 ± 15.7 cm) and mean body fat percentage (32.9% ± 10.8%) were above the recommended values. The total energy intake from drinks was more than a third of the estimated daily energy requirements. Although there was no significant difference in the mean daily caffeine intake, those with affective disorders had a significantly higher intake of sugars from drinks (median (Mdn) = 80.0 (interquartile range (IQR) = 89.8) g/day) and alcohol (Mdn = 45.6 (IQR = 31.1) g/day), compared to those with schizophrenia (Mdn = 60.0 (IQR = 45.4) g/day and Mdn = 24.9 (IQR = 19.8) g/day, respectively).

Conclusions

Considering the link between high sugar and alcohol intake with excess body weight and mental health, these preliminary data are of particular concern and point to the need for better dietary counseling in order to improve the dietary behaviors of these patients.

## Introduction

A major category of mental disorders is affective disorders, which include depressive disorders with or without associated anxiety or hypersomnia. In addition to being an important form of affective disorder, depression is the most common mental disorder [1], with more than 300 million people being affected globally according to the World Health Organization (WHO) [2]. Another category of mental disorders is schizophrenia, schizotypal, and delusional disorders that affect approximately 1% of the population. Schizophrenic disorders are characterized by distortion of thought and perception as well as blunt or flat feeling [1]. In Greece, the prevalence of general psychiatric morbidity of at least mild severity is estimated to be approximately 14% among the general population, with generalized anxiety disorder and depressive episodes being the most common ones [3].

Many studies have suggested the link between diet and mental health [4-6]. Depression has been associated with unhealthy lifestyles, including alcohol drinking and poor dietary patterns. According to a recent review, obesity, metabolic syndrome, and cardiovascular diseases are frequently seen in patients diagnosed with affective disorders, adversely affecting their prognosis [7]. Obesity has been postulated to be both a determinant of depression and an outcome of depression [8].

The role of sugar-sweetened drinks (SSDs) in excessive dietary sugar intakes leading to an imbalance in caloric intake and development of overweight and obesity is of great importance. Common nonalcoholic drinks such as carbonated and fizzy drinks, nectars and fruit juices, sweetened milk and chocolate drinks, sports and energy drinks, and sugar-sweetened coffee and tea can be major contributors to dietary sugar intake. A recent analysis showed that each 1% rise in soft drink consumption was associated with an additional 4.8% increase in overweight in adults [9]. The World Health Organization recommends that sugar intake should be less than 10% of the total energy intake (strong recommendation) and, if possible, less than 5% of the total energy intake (conditional recommendation) [9]. The impact of SSDs on mental health has attracted widespread interest from researchers. SSDs have been associated with a higher prevalence of depression and anxiety [10]. In addition, high sugar intake has also been reported in patients with schizophrenia and can predict the severity of schizophrenia symptoms [6]. Therefore, reduction or abstinence from sugar-containing drinks may be an appropriate public health strategy not only to support those aiming to achieve healthy body weight [11] but also to reduce the risk or severity of depression and anxiety, and schizophrenia symptoms in psychiatric patients. In addition to sugars, alcohol overconsumption may also be tipping the body weight balance in favor of weight gain and increase the risk of obesity.

The association between alcohol dependence/abuse and affective symptoms including depression is well established [12] and prevalent; particularly, those with moderate or severe affective symptoms are more likely to engage in hazardous alcohol use than those with minimal depression. Alcohol is also known to have dramatic effects on the frequency and intensity of psychotic episodes and life expectancy. Interestingly, compared to patients with schizophrenia, patients with depression have been reported to be 11 times more likely to engage in hazardous alcohol use [12,13].

Energy drinks, coffee and tea, and, to a lesser extent, some soft drinks and cocoa/chocolate drinks are common dietary sources of caffeine. There is evidence that caffeine consumption can confer both benefits and risks in a variety of mental disorders depending on the dose [14]. Caffeine has been associated to generate psychostimulant effects through modulating dopaminergic transmission, which is related to depression. High intakes and long-term use of caffeine have been associated with psychiatric disorders [14], triggering psychiatric symptoms such as anxiety, depression, and even psychosis [15]. Symptoms of caffeine overdose toxicity generally include anxiety, insomnia, gastrointestinal disorders, tremor, psychomotor anxiety or arousal, and, in some cases, even death [16]. In addition to causing or exacerbating psychiatric symptoms, caffeine use can interact with many psychiatric medications [17], leading to poisoning due to synergistic effects.

There is limited knowledge regarding the intake of sugars, caffeine, and alcohol among psychiatric patients in Greece. The aim of this study was to evaluate body composition and estimate sugar, alcohol, and caffeine intakes from drinks among outpatients with schizophrenia and affective disorders attending the psychiatric clinic of the University General Hospital of Larissa (UGHL) in Greece.

## Materials and methods

Participants

The study was conducted among 89 outpatients (41 males and 48 females) at a tertiary psychiatric clinic of the University General Hospital of Larissa (UGHL) in Greece. The inclusion criteria included a diagnosis of either a schizophrenia spectrum disorder or an affective disorder. The diagnostic evaluation was based on clinician interviews and medical records. Patients who were unable to communicate were excluded from the study. The study was carried out between June 2019 and August 2019.

Participation in this study was voluntary and anonymous. The trained members of the study team explained the study procedures before obtaining written informed consent from the participants. The study was approved by the Institutional Board of the Technological Educational Institute of Thessaly (protocol number: 3812/ΣΕ1/30-5-2019) and was conducted in accordance with the guidelines laid down in the Declaration of Helsinki.

Anthropometric data

Body weight was measured using a Tanita Body Composition Scale BC-601 (Tanita Health Equipment HK Ltd., East Kowloon, Hong Kong), following the standard procedure (i.e., wearing light clothes and without their shoes). Body height was measured using a Seca 213 Portable Stadiometer (Seca Deutschland, Hamburg, Germany). Body mass index (BMI) was calculated by dividing the body weight (in kilograms) by the square of the height (in meters); a BMI < 18.5 indicates underweight, 18.5-24.9 indicates normal weight, 25-29.9 indicates overweight, and ≥30 indicates obesity. Body fat was assessed using Maltron Body Fat Analyzer BF-900 (Maltron International Ltd., Essex, England). Waist circumference was measured using Seca 203 Measuring Tape (Seca Deutschland, Hamburg, Germany). According to the WHO guidelines, for men, the waist circumference is considered increased if it is >94 cm and significantly increased if it is >102 cm; for women, the waist circumference is considered increased if it is >80 cm and significantly increased if it is >88 cm [18]. Basic metabolism was calculated using the Mifflin-St. Jeor equation, i.e., 10 × weight (kg) + 6.25 × height (cm) - 5 × age (y) + 5 in men and 10 × weight (kg) + 6.25 × height (cm) - 5 × age (y) - 161 in women. The percentage of body fat was calculated in a smaller sample because three patients were unable to complete the examination for medical reasons (i.e., two patients had a pacemaker, and one patient had undergone orthopedic surgery with placement of plates).

Dietary data

The participants were initially asked to complete a previously validated semiquantitative drink frequency questionnaire (DFQ) that uses the “past month” reference period [19]. Although the questionnaire can be self-administered, trained personnel posed the questions and ensured that all questions were answered. The questionnaire consists of 41 questions in seven categories: (i) milk of varying fat and sugar content (six items), (ii) tea and coffee (six items), (iii) fruit juices and nectars (three items), (iv) alcoholic beverages (seven items), (v) soft drinks (six items), (vi) energy drinks (10 items), and (vii) water (three items). No brand names are indicated in the questionnaire, except for caffeinated energy drinks, in order to minimize confusion between these drinks and sports drinks. The participants were asked to indicate the number of days within a typical week that they consumed the specific drink (0-7) and estimate the quantity consumed per day. Quantification of the amounts consumed was facilitated with the use of photographs of typical quantities. The mean intake of each item consumed daily was estimated using the following formula: (frequency × portion size)/7. The estimation of the daily intake of free sugars, alcohol, caffeine, and total calories from the reported drinks consumed was done using the Food Processor Nutrition Program software (version 7.4). Physical activity level (PAL) was also calculated for estimating daily energy requirements.

Statistical analysis

Statistical analysis was performed using SPSS version 20 (IBM Corp., Armonk, NY, USA). Anthropometric characteristics and body fat percentage; daily intake of energy, free sugars, alcohol, and caffeine from drink consumption; and water consumption are expressed as mean and standard deviation (M ± SD) and median (Mdn) and interquartile range (IQR), when appropriate. The Shapiro-Wilk test was used to assess the normality of anthropometric and dietary variables, indicating that distributions may be assumed normal for anthropometric data only. Therefore, for anthropometric data, independent sample t-test was used to assess the differences between groups, while for dietary data, the nonparametric Mann-Whitney U test was used. Pearson chi-square test was calculated for the assessment of the association between categorical variables. For the results of the t-test, Mann-Whitey U test, and chi-square test, effect sizes were reported to assess the magnitude of each bivariate association. Then, with the purpose of assessing possible relationships between dietary data and mental disorders, binary logistic regression was utilized, setting the schizophrenia group as the reference category. Independent variables were examined for collinearity issues with the variance inflation factor (VIF < 2.5 according to Johnston et al. (2018) [20]). Two models were produced, the first only with dietary data as independent variables (energy intake from nonalcoholic drink consumption, free sugar intake, caffeine intake, water consumption, and alcohol intake) and the second with dietary data while adjusting for anthropometric characteristics (gender, age, and waist circumference). For all comparisons, the significance level was 0.05.

## Results

A total of 89 participants (60% with affective disorder and 40% with schizophrenia) participated in this study. The anthropometric characteristics of the participants are depicted in Table 1. Those with affective disorders were of higher mean age (d = 0.535, p = 0.006) and slightly higher waist circumference (d = 0.078, p = 0.032) compared with those suffering from schizophrenia. No significant differences were observed for BMI or body fat percentage between those suffering from affective disorders and those suffering from schizophrenia.

**Table 1:** Anthropometric characteristics and body fat percentage in participants with affective disorders and schizophrenia. The results are presented as means ± SD, and p-values were calculated with an independent sample t-test. *Effect size of t-test expressed with Cohen’s d. **Effect size of chi-square test expressed with Phi. ***Three patients were unable to complete the examination of bioelectrical impedance for medical reasons.

	Total (N = 89)	Affective disorders (N = 53)	Schizophrenia (N = 36)	p	Effect size
Age (years)	47.3 ± 12.7	50.4 ± 14.0	42.89 ± 9.15	0.006	0.535*
Number of females (%)	48 (53.9%)	33 (62.3%)	15 (41.7%)	0.056	0.203**
BMI (kg/m^2^)	28.9 ± 5.6	29.5 ± 5.9	28.12 ± 5.0	0.250	0.235*
Waist circumference (cm)	102.6 ± 15.7	103.1 ± 15.8	101.9 ± 15.7	0.032	0.078*
	Total (N = 86***)	Affective disorders (N = 50)	Schizophrenia (N = 36)		
Body fat (%)	32.9 ± 10.8	34.4 ± 11.1	31.0 ± 10.3	0.159	0.304*

Females were significantly older compared to males (50.8 ± 13.5 versus 43.3 ± 14.0, respectively) (p = 0.005) and had a significantly higher percentage of body fat (38.41% ± 9.13% (females) versus 26.37% ± 8.64% (males)) (p < 0.001). However, there were no significant differences between genders in mean BMI (29.7 ± 5.8 kg/m^2^ (females) versus 28.1 ± 5.2 kg/m^2^ (males)) (p = 0.186) and waist circumference (102.3 ± 13.9 cm (females) versus 102.9 ± 17.7 cm (males)) (p = 0.88). In men, the percentage of body fat among those with affective disorder was 26.5 ± 8.4 and among those with schizophrenia was 26.7 ± 9.0 (p = 0.866). In women, the percentage of body fat among those with affective disorder was 38.7 ± 9.6 and of those with schizophrenia was 37.7 ± 8.2 (p = 0.673).

**Figure 1:**
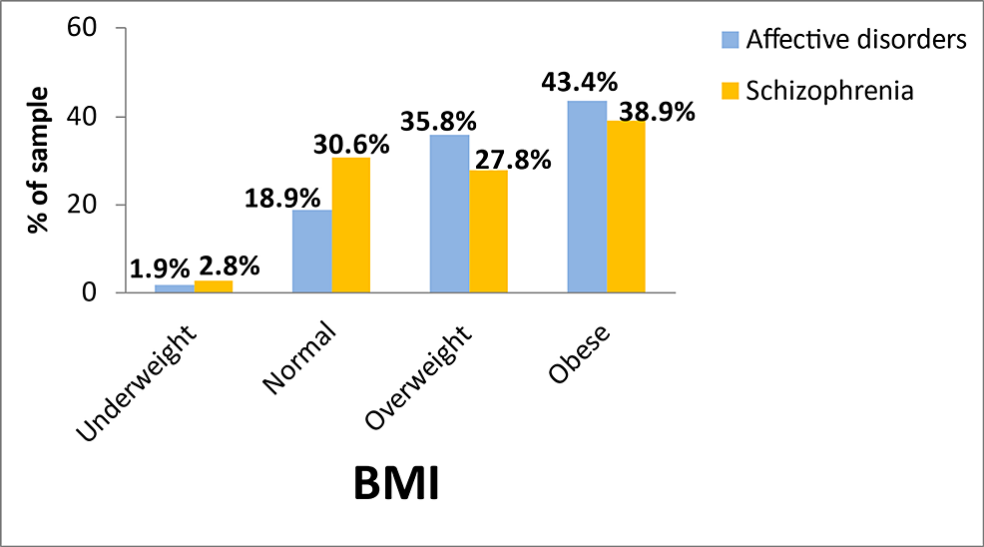
Percentage of patients with affective disorders and schizophrenia in different BMI categories.

**Figure 2:**
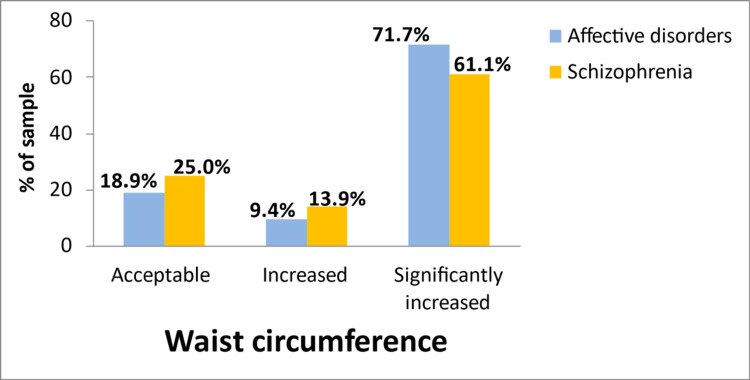
Percentage of patients with affective disorders and schizophrenia according to different waist circumference cutoff points (i.e., increased: >80 cm for females and >94 cm for males; significantly increased: >88 cm for females and more than 102 cm for males).

With regard to BMI, almost 80% of those with affective disorders and 67% of those with schizophrenia were overweight or obese (Figure 1). In addition, only 19% of patients with affective disorders and 25% with schizophrenia had recommended waist circumference (i.e., <80 cm for females and <94 cm for males) (Figure 2). The percentage of fat was higher than the recommended values of the American Council on Exercise (i.e., >18% for males and >25% for females) [21] in 88% of patients with affective disorders and 83% of those with schizophrenia (detailed data not shown in tables).

Dietary data

**Figure 3:**
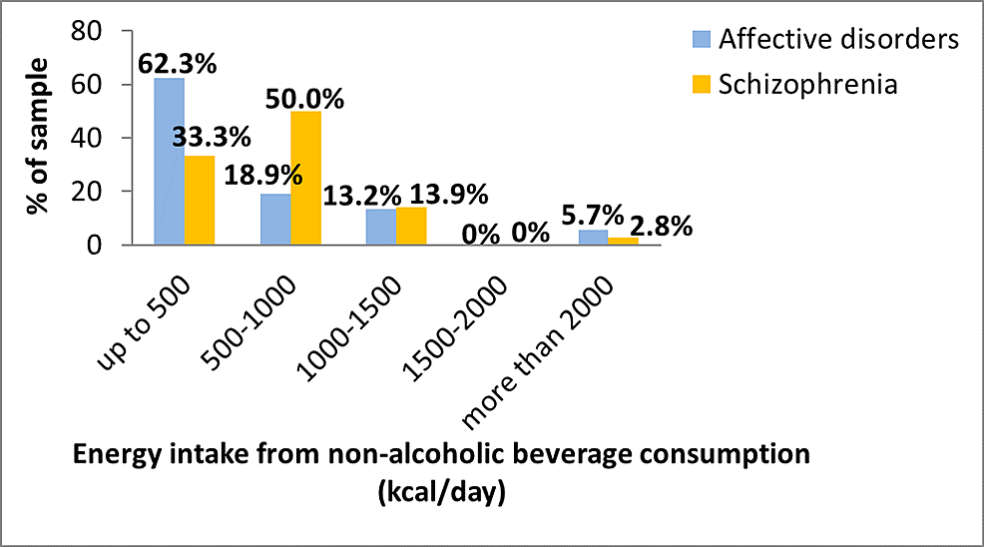
Energy intake from nonalcoholic beverage consumption.

Data related to daily energy, sugars, alcohol, and caffeine intake from drinks are presented in Table 2. The median estimated energy requirement was 1714.24 (IQR = 581.00) kcal/day for those with affective disorders, while the median energy intake from drinks was 543 (IQR = 727.49) kcal/day, i.e., 31.68% of total energy requirements. Similarly, the mean estimated energy requirement for those with schizophrenia was 1657.50 (IQR = 422) kcal/day, while the calculated median energy intake from drinks was 783.42 (IQR = 580.67) kcal/day, i.e., 47.7% of total energy requirements. Although there were no significant differences in the median values of energy intake from total drink consumption, those with schizophrenia presented a significantly higher energy intake from nonalcoholic drinks compared with those with affective disorders (r = 0.209, p = 0.048) (Figure 3), while those with affective disorders had a significantly higher median intake of sugars from drinks than those with schizophrenia (r = 0.274, p = 0.010) (Table 2). No significant differences were found between males and females, in both groups of patients, about energy intake from both nonalcoholic and all drinks, or sugar intake from drinks (p > 0.05).

**Table 2:** Daily intake of energy, free sugars, alcohol, and caffeine from drink consumption. The results are presented as median and interquartile range (IQR) values, and p-values were calculated using the Mann–Whitney U test. *Alcohol consumers only.

	Total (N = 89)	Affective disorders (N = 53)	Schizophrenia (N = 36)	p	r
Energy intake from total drink consumption (kcal/day)	618.22 (703.20)	543.00 (727.49)	783.42 (580.67)	0.067	0.194
Energy intake from nonalcoholic drink consumption (kcal/day)	505.00 (617.27)	416.10 (628.13)	654.00 (533.18)	0.048	0.209
Free sugar intake (g/day)	69.00 (62.50)	80.00 (89.80)	60.00 (45.40)	0.010	0.274
Caffeine intake (mg/day)	162.80 (174.70)	150.30 (133.30)	187.81 (204.20)	0.227	0.128
Water consumption (mL/day)	1750 (1375)	2000 (1500)	1500 (1000)	0.007	0.284
	Total (N = 40*) (44.9% of the total sample)	Affective disorders (N = 23*) (43.4% of affective disorder sample)	Schizophrenia (N = 17*) (47.2% of schizophrenia sample)		
Alcohol intake (g/day)*	33.60 (27.04)	45.56 (31.11)	24.87 (19.77)	<0.001	0.576

In relation to alcohol intake, 43.4% of those with affective disorders and 47.2% of those with schizophrenia reported alcohol consumption, with those suffering from affective disorder reporting significantly higher alcohol consumption than those with schizophrenia (p < 0.001) (Table 2). In addition, 41.5% of patients with affective disorders and 33.3% of patients with schizophrenia exceeded the alcohol intake recommendations for the general public (i.e., 10 g/day for females and 20 g/day for males) [22].

With regard to caffeine intake (Table 2), no significant differences were found between males and females in both groups of patients (affective disorders, p = 0.073; schizophrenia, p = 0.268). Among those with affective disorders, six participants (11.3%) exceeded the European Food Safety Authority (EFSA) recommendations of <400 mg/day for caffeine [23] and had a mean caffeine intake of 638.4 ± 241.2 mg/day. In addition, among those with schizophrenia, five participants (13.9%) exceeded the EFSA recommendations and had an estimated mean caffeine intake of 597.7 ± 187.9 mg/day. Although men had a higher mean caffeine intake (239.8 ± 195.7 mg/day) compared with women (206.2 ± 178.6), the difference was not significant (p = 0.401).

Finally, patients with schizophrenia consumed significantly less water (Mdn = 1500 mL/day, IQR = 1000 mL/day) than patients with affective disorders (Mdn = 2000 mL/day, IQR = 1500 mL/day) (p = 0.007). No significant difference was detected between genders in water consumption. More specifically, among women with affective disorders, 39.4% reported water consumption of ≤1000 mL/day, while among women with schizophrenia, 73.3% reported water consumption of ≤1000 mL/day. For men, 55% reported less than recommended water consumption in the affective disorder group and 76.2% in the schizophrenia group.

In order to examine the effects of dietary data on the classification of patients in the two groups (affective disorders versus schizophrenia), two logistic regression models were calculated, using the dietary and anthropometric data as independent variables. The variables BMI, body fat, and energy intake from total drink consumption were not included in the models due to collinearity issues. When examining the effects of dietary data only (Table 3, unadjusted model), energy intake from nonalcoholic drinks significantly decreased the odds of belonging to the affective disorder group compared to schizophrenia, for both energy intake levels (501-1000 versus <500 kcal/day: odds ratio (OR) = 0.044 (95% confidence interval = 0.009, 0.222) (p < 0.001); >1000 versus <500 kcal/day: OR = 0.043 (95%CI = 0.005, 0.377) (p < 0.01)), while free sugar intake significantly increased the odds of affective disorders compared with schizophrenia for both consumption levels (50-100 versus <50 g/day: OR = 5.482 (95%CI = 1.242, 24.207) (p < 0.001); >100 versus <50 g/day: OR = 68.208 (95%CI = 8.223, 565.744) (p < 0.05)). However, when adjusting for demographics and anthropometric characteristics (Table 3, adjusted model), energy intake from nonalcoholic drinks significantly decreased the odds of affective disorders compared with schizophrenia only for 501-1000 kcal/day consumption level (OR = 0.038 (95%CI = 0.007, 0.214) (p < 0.001)), and free sugar intake significantly increased the odds of affective disorders compared with schizophrenia only for >100 g/day consumption level (OR = 165.4 (95%CI = 13.51, 2025) (p < 0.001)). Age significantly increased the odds of affective disorders compared with schizophrenia in this sample (OR = 1.108 (95%CI = 1.017, 1.207) (p < 0.05)). Therefore, it can be concluded that dietary characteristics such as energy intake from nonalcoholic drink and free sugar intake may independently predict psychiatric disorders (affective disorders versus schizophrenia).

**Table 3:** Logistic regression results (odds ratio and 95%CI) for the classification of patients in the affective disorder group versus schizophrenia group according to dietary data and anthropometric characteristics. ^1^ More than 10 g/day for females and 20 g/day for males ^2^ More than 80 cm for females and 94 cm for males ^3^ More than 90 cm for females and 100 cm for males

	Affective disorder group versus schizophrenia group			
	Unadjusted model		Adjusted model	
	OR (95%CI)	p	OR (95%CI)	p
Energy intake from nonalcoholic drinks				
501–1000 versus <500 kcal/day	0.044 (0.009, 0.222)	<0.001	0.038 (0.007, 0.214)	<0.001
>1000 versus <500 kcal/day	0.043 (0.005, 0.377)	0.005	0.09 (0.008, 1.015)	0.051
Free sugar intake				
50–100 versus <50 g/day	5.482 (1.242, 24.207)	<0.001	4.856 (0.99, 23.823)	0.052
>100 versus <50 g/day	68.2 (8.223, 565.744)	0.025	165.4 (13.51, 2025)	<0.001
Caffeine intake > 400 mg/day	0.589 (0.099, 3.496)	0.560	0.617 (0.101, 3.766)	0.601
Water consumption < 1000 mL/day	0.299 (0.092, 0.972)	0.045	0.375 (0.106, 1.327)	0.128
Alcohol intake (increased)^1^	0.886 (0.266, 2.954)	0.844	0.641 (0.167, 2.465)	0.518
Gender (female versus male)			1.306 (0.31, 5.495)	0.716
Age (years)			1.108 (1.017, 1.207)	0.019
Waist circumference (cm)				
Increased^2^			0.804 (0.119, 5.43)	0.823
Significantly increased^3^			1.347 (0.248, 7.307)	0.730

## Discussion

The findings of this pilot study provide important insights into the consumption of drinks as reported by outpatients from a psychiatric clinic in Greece. Drink consumption habits and body composition were evaluated in 89 Greek outpatients with mental disorders. Our results show that the majority of participants were overweight or obese. Drinks contributed more than one-third of the estimated total daily energy requirements, with sugar intake exceeding the WHO recommendations from drinks alone. This was particularly apparent among those with affective disorders. Less than half of the participants had alcohol intakes exceeding the guidelines for healthy adults, while caffeine intake did not seem to be of major concern for the majority of the participants.

Sugars

In line with previous research [11,14,24], increased consumption of sugars from drinks was observed among patients with affective disorders and schizophrenia, exceeding in both groups of patients the WHO guidelines for sugar intake from all dietary sources [9]. Sugar intake from drinks among those with affective disorders (mainly depressive disorders) was found to be higher than the amount estimated among those with schizophrenia. High sugar intake has been previously reported to play a causal role in the risks of both incident and recurrent depression [8]. The link between high sugar intake and the risk of depression may indeed have a biological basis. In particular, a high sugar diet may decrease brain-derived neurotrophic factor (BDNF) levels and increase circulating inflammatory markers, which may depress mood. Postprandial hypoglycemia due to an exaggerated insulin response following a high sugar intake, as well as the addiction-like effects of sugars influencing dopaminergic neurotransmission mechanisms, may also provide an explanation about the link between high sugar intake and low mood [8]. Consumption of soft drinks sweetened with sugar has been positively associated with the prevalence of major depressive disorder history diagnosis in a multicountry sample of overweight participants with subsyndromal depressive symptoms [25]. Furthermore, overconsumption of sugars promotes long-term dysregulation of the stress response. In particular, the activity of the hypothalamic-pituitary-adrenal (HPA) axis, which is known to partially regulate stress response, has been shown to be reduced after consumption of sugar-containing foods [26]. In our study, although patients with schizophrenia had lower sugar consumption compared with those with affective disorders, their sugar intake from drinks alone was still regarded as high. This is a cause of concern since a diet high in refined sugars has been shown to predict a worsening of schizophrenic behavior [27]. Interestingly, while early research on addiction has focused on substances of abuse such as alcohol, this has since been extended to include more recently sugar consumption. However, there are currently no therapies directed at reducing sugar consumption [28]. Hence, more focused research on novel pharmacotherapeutics and/or other interventions is needed to reduce sugar intake among these patients.

Excessive intake of sugars and energy from drinks has been shown to increase the risk of obesity, which in turn may be associated with mental health disorders. According to their BMI, most patients were categorized as overweight/obese, a finding which is in accordance with other studies that include patients with mental disorders [11]. Although BMI and the percentage of body fat were not statistically different among the two groups of psychiatric disorders, those with affective disorders tended to have higher waist circumference than those with schizophrenia, indicating greater accumulation of abdominal fat, which is typically seen among those with metabolic syndrome. This finding is also parallel to the trend seen in the sugar intake from drinks. Indeed, there is evidence supporting a pathological predisposition to metabolic syndrome in both depression and schizophrenia, which can explain the higher incidence of metabolic syndrome seen in these patients compared to the general population [29]. Sugar-containing drinks may, at least partly, be responsible for excessive weight and obesity, while obesity can be associated with the development of depression not only via inflammatory but also psychosocial factors such as weight discrimination [11,30]. Previous research has indicated that consumers of sugary drinks often have lower levels of health awareness and are more likely to underestimate the sugar content of a sugar-containing drink than nonconsumers [24]. These preliminary data, therefore, point to the need for better nutritional counseling in order to improve their eating behaviors, particularly among those with affective disorders.

Caffeine

Caffeine consumption in our sample was not high, with approximately only one in 10 of the participants exceeding the EFSA recommendations for the general public. Navarro et al. (2018) found that participants who consumed at least four cups of coffee per day showed a lower risk of depression than participants who drank less than one cup of coffee per day [31]. They also reported no significant dose-response relationship between coffee consumption and the risk of depression. Moreover, a recent study showed that black tea consumption up to four cups and a caffeine intake between 450-600 mg/day can help protect against depression [32]. In our study, the median caffeine intake was estimated to be less than that (approximately 160 mg/day). Any protective effect of caffeine in patients with affective disorders is unclear in our study. This could be further investigated in future studies. With regard to schizophrenia, high caffeine intake can result in an increase in dopamine activity, which may, in turn, worsen positive symptoms of schizophrenia such as hallucinations and delusions [33]. It has been reported that patients with schizophrenia consume twice as much caffeine on average when compared to healthy controls [34], a finding that was not observed in our study for the majority of patients with schizophrenia. On the other hand, plenty of preclinical and clinical evidence suggests that caffeine consumption could also have a beneficial effect on schizophrenia, depending on a number of factors, including symptoms, gender, and activity of metabolic enzymes [35]. While moderate caffeine (≤250 mg/day) appears to result in better performance on cognitive tasks in schizophrenic patients [36], more research is needed to determine whether the caffeine recommendations in this population group are the same as that of the general healthy population.

Alcohol

Patients with affective disorders reported higher alcohol consumption than those with schizophrenia. These results are consistent with other studies, suggesting a positive association between alcohol consumption and obesity [11], as well as schizophrenia and affective disorders [12]. Alcohol dependence and affective disorders are frequently comorbid with varying prevalence and share underlying mechanisms [37]. Excessive alcohol consumption can worsen the depression course, increase the risk of suicide, and delay recovery from psychiatric conditions [30]. With regard to schizophrenia, a study conducted in patients with schizophrenia in Singapore showed that those with greater symptom severity were approximately twice as likely to have problematic alcohol use [30]. A case-control study similarly reported a higher number of patients with schizophrenia who had problematic alcohol consumption as compared to matched controls from the general population [38]. In our study, less than half of the sample of patients with schizophrenia or affective disorders reported alcohol use, and among users, about four in 10 of those with affective disorders and about one in three with schizophrenia exceeded the recommendations for healthy adults. According to the American Heart Association, moderate alcohol consumption is about two glasses/day (20 g/day of alcohol) for men and one glass per day (10 g/day of alcohol) for women [22]. In our study, despite the relatively low number of patients with affective disorders or schizophrenia who might be at risk of alcohol overconsumption, clinicians need to be vigilant to detect early any problem use of alcohol. Health professionals should focus on ways to empower these patients to minimize the consumption of alcoholic drinks that can worsen their symptoms and contribute to weight gain due to the empty calories they provide [11].

Limitations of the study

This study is cross-sectional, and hence, no causality or direction could be inferred from our data. There was no information about prognosis or symptom severity that could provide insights on whether these dietary factors worsen the affective disorders or schizophrenia symptoms. No control group from a healthy sample was included to infer any differences between patients with psychiatric illness and healthy controls. Dietary data were obtained through self-reporting, which may be subject to recall bias and/or social desirability bias. No information on the medication prescribed was collected, which could provide some possible explanation on the trends seen in drink consumption patterns. Since this study is a pilot study, the sample size was relatively small, thus limiting the generalizability of the current results. In light of the high intake of sugars from drinks alone, in future studies, it would be interesting to collect detailed food consumption data via diet diaries or 24-hour dietary recalls that could capture the consumption of solid foods high in sugars such as chocolate, cakes, and biscuits. Further studies are needed to confirm our preliminary results and investigate any confounding variables. There is a need for longitudinal studies to evaluate the association between dietary, anthropometric and symptom severity data and prognosis, as well as meta-analysis to support any food-based dietary guidelines specific for these patients.

## Conclusions

In our study, sugar consumption from drinks was high particularly among patients with affective disorders rather than those with schizophrenia. A similar association was evident with alcohol consumption with those diagnosed with affective disorders consuming greater amounts compared with those with schizophrenia, although the percentage of alcohol users between the two groups was not significantly different. Such findings may partly explain the higher waist circumference seen in those with affective disorders compared with those suffering from schizophrenia. A relatively small number of the sample exceeded the recommended caffeine daily intake. Further well-designed large prospective studies are needed to provide definitive evidence to address the effects of various types of drinks on the risk of affective disorders and schizophrenia. Our results suggest the need for more research into psychotherapeutic and pharmacological interventions to improve drink consumption habits and reduce the intake of sugars and alcohol among these types of psychiatric patients.
